# Fifteenth century Florentine mural investigated in situ with VNIR Hyperspectral Imaging and NIR Photography supports interpretation as a bloodletting scene

**DOI:** 10.1038/s41598-024-58972-1

**Published:** 2024-05-22

**Authors:** Costanza Cucci, Simon Donell, Elisa Zucchini, Marcello Picollo, Lorenzo Stefani, Donatella Lippi

**Affiliations:** 1 Institute of Applied Physics, “Nello Carrara” - National Research Council (CNR-IFAC), Via Madonna del Piano, 10, 50019 Florence, Italy; 2 Norwich Medical School, University of East Anglia, Norwich, NR4 7TJ UK; 3 Department of History, Archaeology, Geography, Art and Performance (SAGAS), University of Florence, Via S. Gallo, 10, 50129 Florence, Italy; 4 Department of. History of Medicine and Medical Humanities, University of Florence, P.Zza S. Marco, 4, 50121 Florence, Italy

**Keywords:** Remote sensing imaging, Visible and near infrared (VNIR) hyperspectral imaging, Near Infrared (NIR) photography, Mural painting, Non-invasive investigations, Buonomini, Florence San Martino Oratory, Bloodletting, Medical history, Imaging techniques, Health care

## Abstract

This study provides new data which suggest a novel interpretative hypothesis not only on the specific painting, but on the use of bloodletting as medical practice in the Florentine Quattrocento. As a part of a cycle of frescoes devoted to the Seven Corporal Works of Mercy, the examined lunette depicts the “Visit to the sick” in a domestic interior, but it has never been considered as an historical document of precise medical practices. The scene’s definitive interpretation is still unresolved because of the uncertainty of some iconographic details. A campaign of in-situ and non-invasive technical investigations was performed to retrieve possible traces of previous details today concealed. The technical solutions adopted to implement the measurements campaign are illustrated, as an experimental example for remote sensing inspection of mural paintings in-situ. The position of the painting high up on a wall of an historical venue led to opting for stand-alone optical imaging techniques which could operate in remote sensing mode. By combining the use of portable Hyperspectral Imaging with Near Infrared photography a set of detailed images could be obtained that highlighted details not otherwise detectable. Focused on the objects held by the persons present, the analysis of the mural of *Visit of the Buonomini in her Lying in Bed*, the gift of swaddling cloth could be a tourniquet, shadows of folds of a blanket a thumb lancet, and an object held a blood collection bowl, supported the hypothesis that it could be a medieval bloodletting scene.

## Introduction

Science and Art are intimately interlinked by the materials used and the production of the work; as science and technology advance so do the arts. Scientific analysis of artworks provides art historians with additional data (materials, dating, conservation status, provenance, etc.)^1^. Analysis and interpretation of paintings and figurative arts can be crucial for many fields of knowledge including medicine, science, and socio-economics. The interlinking of different methodological and disciplinary approaches allows triangulation of historical material thus evaluating both the subject of each picture and its contextualisation, characteristics, and conservation state. Technical investigations of paintings are a crucial step not only for preservation and for their understanding as works of art, but also when they are meant as historical visual documentation in multidisciplinary research^2^. Several technologies are today available for analysing artworks, and notably paintings^3–5^. The choice of the methodologies and experimental protocols is primarily determined by the research question, but it might be also constrained by financial resources, feasibility, or logistic difficulties. Non-invasivity is always a prerequisite, and often a mandatory requirement. Recent research has demonstrated the effectiveness of multi-modal imaging for non-invasive inspection of the entire painting surface and its hidden features by combining advanced imaging techniques, such as Reflectance Imaging Spectroscopy in the Visible, Near-Infrared and Short-wave Infrared (VNIR-SWIR) ranges and Macro-X-rays Fluorescence (MA-XRF)^6,7^. Nevertheless, these methodologies use high-precision scanning devices and cumbersome instrumentation which usually operate at close range from the targets and require controlled set-ups^8^. Therefore, while this approach is ideal for a comprehensive analysis of panel paintings and manuscripts, it is unfeasible for other types of artefacts, such as mural paintings, which cannot be moved and may be awkwardly located. Even if today’s methods for diagnosing cultural heritage are very sophisticated, non-invasive analysis of wall paintings still remains a challenging task. This is not only because murals feature big sizes and are usually located in places hard-to reach (lunettes, caves, ceilings, etc.), but also because they are complex physico-chemical systems in terms of materials, manufacturing processes, and degradation products. Thus, high variability of materials and conservation state is often observed over the surface that results in a greater need of data for statistical significance. Portable devices for in-situ measurements can respond to this exigency, provided that the representative areas of the mural can be measured, for example using scaffolding. Over the last decade, an increased number of technical studies on wall paintings have been reported^9^. Several analytical methods, in particular spectroscopic techniques, such as X-ray fluorescence (XRF), Raman spectroscopy (RS), Fourier Transform Infrared (FT-IR), diffuse reflectance infrared (DRIFTS), energy-dispersive X-ray fluorescence (EDXRF), only to list some of the most well-established ones, are used for identifying pigments, binders, and some deterioration products^10–13^. These measurements are performed in-situ on a selection of points, or performed in laboratory on detached fragments. To extend the information and document the wider areas of the painting, imaging techniques are required. The imaging mode is crucial whenever the task is the examination of the figurative arts. Indeed imaging techniques not only enable extensive investigation of surfaces rather than single points, but also enable visualisation of the original surface as elaborated views, or false colour images or maps where elements that cannot be appreciated visually are highlighted in the targeted scene. A typical example is IR imaging displaying underdrawings or covered traits under the visible painted layers.

Up to now, the most well-established approach involves cooperative use of imaging techniques operating in different regions of the electromagnetic spectrum, in order to enable inspection of the surface at different penetration depths. For example, a masterpiece fresco by Beato Angelico has been fully examined from the outer painting surface towards the inner support by combining Ultraviolet fluorescence (UVF), Infrared Reflectography (IRR), TeraHertz (THz) imaging, and Ground Penetrating Radar (GPR) along with high-resolution Vis digital photograph^14^. One of the most cutting edge methods for frescoes investigations today is THz imaging which has proven to be usable for non-invasive reconstruction of paints stratigraphy, and when is used in combination with VNIR-SWIR hyperspectral imaging, it provides comprehensive characterisation of the pictorial materials (Vis and THz), underdrawings (NIR) and support anomalies (THz)^15,16^. Nevertheless, these experimental protocols can be adopted into the study of important masterpieces, within the framework of large research projects, since they involve cutting-edge and resource-consuming methodologies which require scaffolding. In a few other situations, when the minor figurative arts are under examination, simpler technical approaches may be required.

In this instance stand-alone optical imaging may provide a flexible and minimal impact alternative that allows cost-effective and quick in-situ use. Optical techniques exploit non-ionising electromagnetic radiation, from the Ultraviolet (UV) through the Visible (Vis) up to the Infra-Red (IR) regions, and are non-invasive. Stand-alone optical imaging for cultural heritage applications includes several contactless and remote sensing techniques, which broadly range from more conventional methods (digital and multi-band photography)^17^, to imaging spectroscopy techniques (multispectral and hyperspectral imaging)^18–22^, and the 2D/3D methods of cultural heritage digitisation^23–27^. All these techniques feature high versatility and can be operated in-situ, using minimal supporting equipment. As a primary effect, their use enables the examination of a wider range of artworks. A drawback of stand-alone imaging methods is that the quality of the data might be affected by the measurement set up and operating conditions, especially in difficult spaces. A multimodal approach with a combination of techniques tackles this problem and may partially compensate the potential errors in non-ideal operating conditions^28^. On a case by case basis different methodologies and technical solutions can be tried to answer specific research question^22^. This is the case when analysis of lesser figurative arts becomes important because of their historical context conveying a wider message besides their artistic meaning. For example, the history of medicine builds, not only on ancient treatises and literary documents, but also strongly relies on the examination of visual sources that include a variety of different types of artworks or pictures. Some may illustrate illness, its perception, and its treatments through the ages and through past societies^29–33^.

The present work illustrates how advanced imaging investigations of a minor historical site provided an indirect yet significant record of an undocumented practice of bloodletting in the context of caregiving.

**Figure 1:**
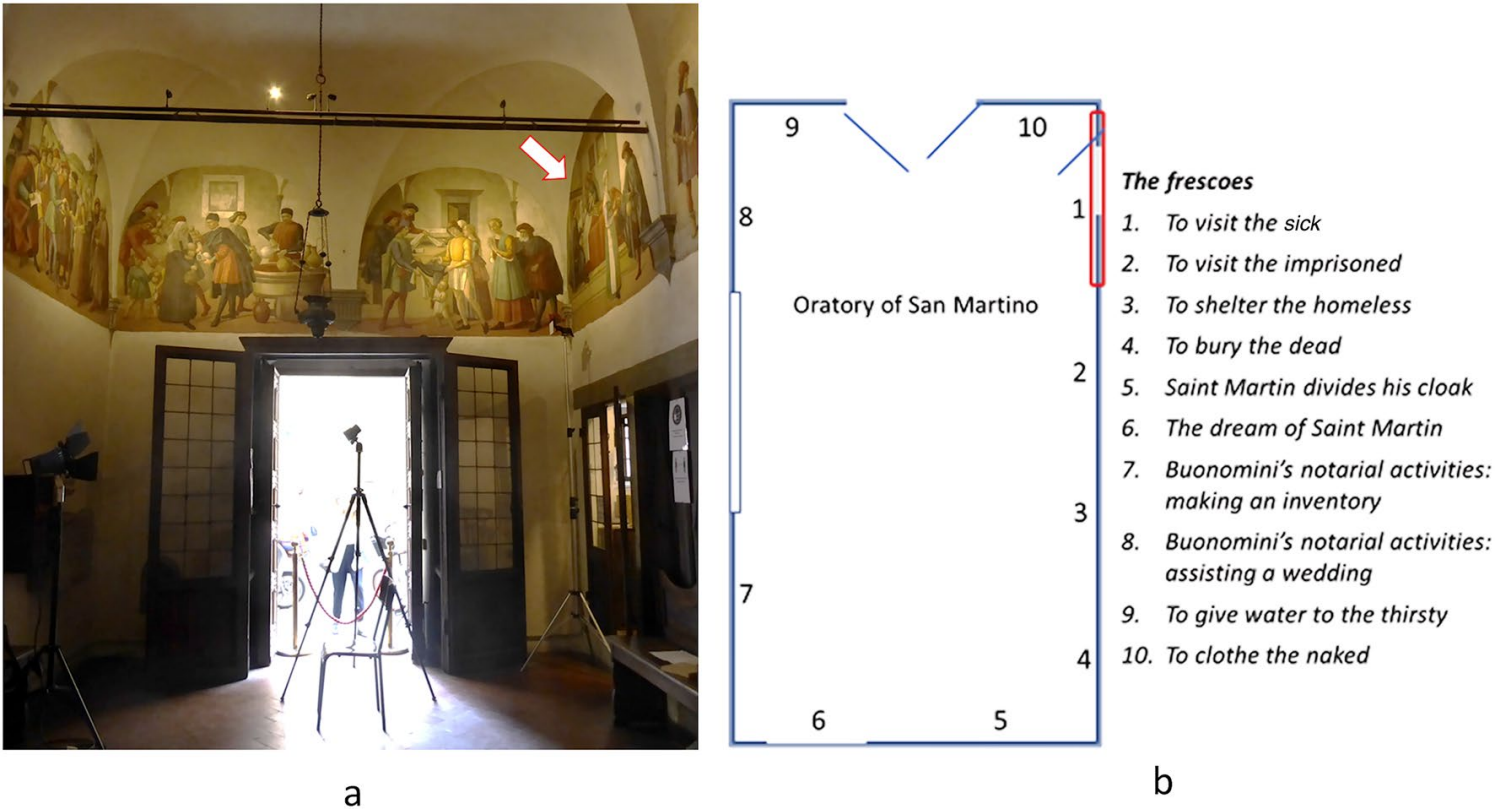
The San Martino Oratory within the church San Martino al Vescovo in Florence. (**a**) View of the frescoed walls (ca 1486 and 1490, Ghirlandaio workshop attrib.) with the location of the examined lunette within its setting. The picture shows the operational environment during the measurement campaign (August 2022). (**b**) Plan of the Oratory room.

**Figure 2:**
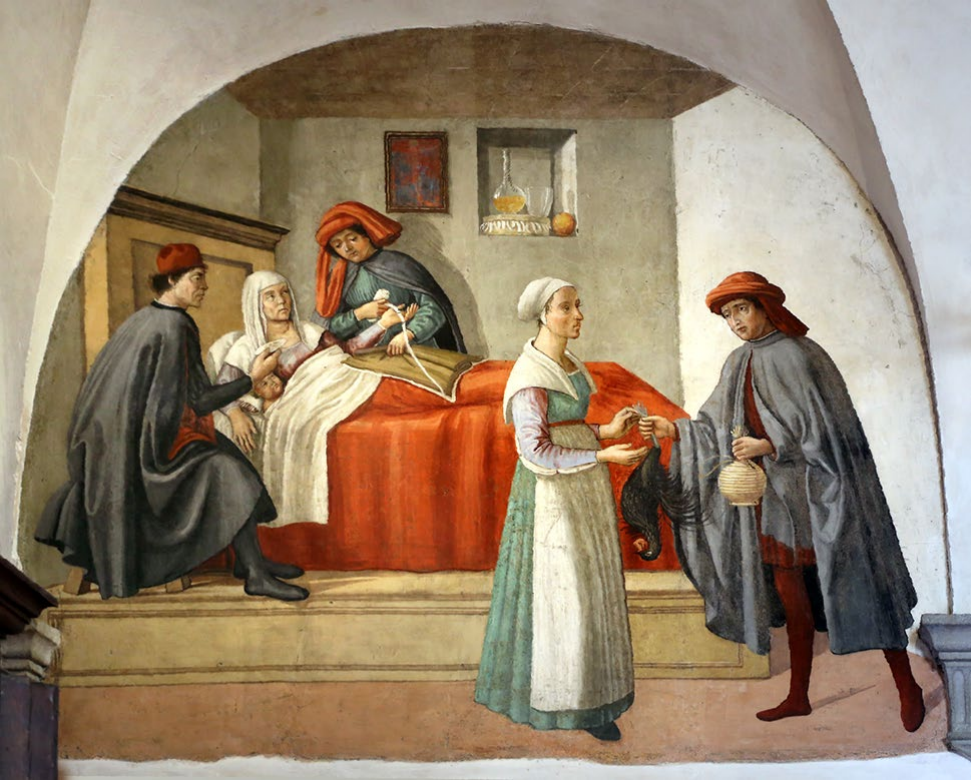
The lunette hosting the painting *Visit of the Buonomini to a Woman in her Lying in Bed* (ca 1486 and 1490, Ghirlandaio workshop attrib.). [Photo Credits: License CC-BY-3.0 by Sailko—Supported by Wikimedia CH].

Around 1480, the Oratory of San Martino became the venue of the Confraternity “I Buonomini di San Martino” (Good Men of St. Martin), a voluntary association which provided assistance to well off people who had fallen on hard times (the “shamed poor”)^34^. It was an important part of the welfare system of Florence. The Oratory walls were decorated with frescoes depicting the story of St. Martin of Tours and scenes of the seven Works of corporal Mercy (Fig. 1). It is generally accepted that the murals were executed by Ghirlandaio’s (1448–1494) workshop, but they are nevertheless recognised as of lesser artistic quality compared to the coeval Ghirlandaio’s cycle in Santa Maria Novella. Their value lies in their historical depiction of Quattrocento Florentine life of ordinary people in their usual environment rather than wealthy or well-known persons^35^. The object of this study is a scene of illness treatment and assistance. The first lunette on the south side wall of the Oratory is devoted to the fifth corporal Work of Mercy, the “Visit to the Sick”*,* titled *Visit of the Buonomini to a Woman in her Lying in Bed*. The painting fills a semi-circular space within the vault of the ceiling in a corner of the room (Fig. 1a). It stands over a stone threshold of an internal doorway making it difficult to position tripods and equipment below the painting or to use of scaffolding. The base of the lunette is 2.10 m wide and is 2.67 m above the floor. The height of the painting at the top of the lunette is about 2 m. The scene (Fig. 2) is in a Florentine room of a modest socioeconomic house^36^. A woman with her swaddled baby lies in a bed and is assisted by two men. Their clothes indicate they belong to the Buonomini Confraternity. The two men are positioned on the opposite sides of the bed. Both are helping the woman in a way that hitherto has been interpreted as an offering of swaddling bands. The standing man holds an unrolled white bandage in his right hand, while with his left hand he seems to handle a brown linen cloth, or perhaps grasping what appears as a fold in the swaddling blanket^37,38^. The gesture seems odd and the action unclear. On the other side of the bed the Buonomo who sits on a chair is differently dressed, thus likely from a higher social level. He holds a small white container in his right hand that is offered to his colleague. On the opposite side of the room, another man stands passing wine and a chicken to a young standing woman. Although the scene is named to as representing the “Visit to the Sick” giving sustenance and clothing to help mother and child, the painting has never been considered by medical historians. The two most likely diagnoses for the woman are *post partum* depression and puerperal fever. Both were treated by bloodletting from the dorsal vein (*salvatella*) of the left hand between the little and ring metacarpals. This vein was used for treating disorders of the spleen which included an excess of black bile or black choler, melancholy^39,40^ or fevers^41^. However, the correct interpretation of the painting remains unresolved due to a lack of definition of some iconographic details. The European therapeutic tradition dates from Hippocrates and the belief in the four humours. This lasted for over 2000 years and continues in Traditional Persian Medicine today^42^. An imbalance of the four humours explained all illnesses. Blood was hot and wet and, if in excess, removing some would bring it back into balance. Bloodletting could be prophylactic to keep the humours in balance, or to treat diseases. The tourniquet was applied to the wrist. Logically this leads to the supposition that the scene in “Visit to the Sick” is about bloodletting. This interpretation of the scene has never been considered until now. If this is the case, then the swaddling cloth is a bandage used as a tourniquet about to be applied to the left wrist, the sitting man is holding a phlebotomy bowl, and the standing Buonomo holds a lancet in his left hand. However this interpretative doubt arises from a loss of definition of some small details, namely the objects in the hands of the two men assisting the woman and the swaddling clothes of the baby. Retrieving the visability of details could clarify the purpose of the objects and shed light of the type of care being given. The current loss of detail may have occurred due to an originally poor executive pictorial technique, but the painting may also have undergone minor changes or misinterpretation during numerous undocumented historical restorations, as is often the case in old lesser cultural sites. Indeed, while the recent restorations (1970 and 2011) have been correctly documented, the murals feature traces of several undocumented repaintings undertaken over the centuries^43^.

The aim of the technical study was to examine the underpainting of “Visiting the Sick” using stand-alone optical imaging techniques, in order to investigate the presence of possible hidden details of the scene and then correctly interpret the represented action. The measurements campaign specifically focussed on providing image documentation usable to confirm or discard the hypothesis that it is a bloodletting scene that shows the Buonomini had acquired the skills to provide medical care. Beyond a revisitation of this specific painting, the proposed interpretation would offer new clues about uses and practices of bloodletting in the Italian Renaissance age.

## Results and discussion

**Figure 3:**
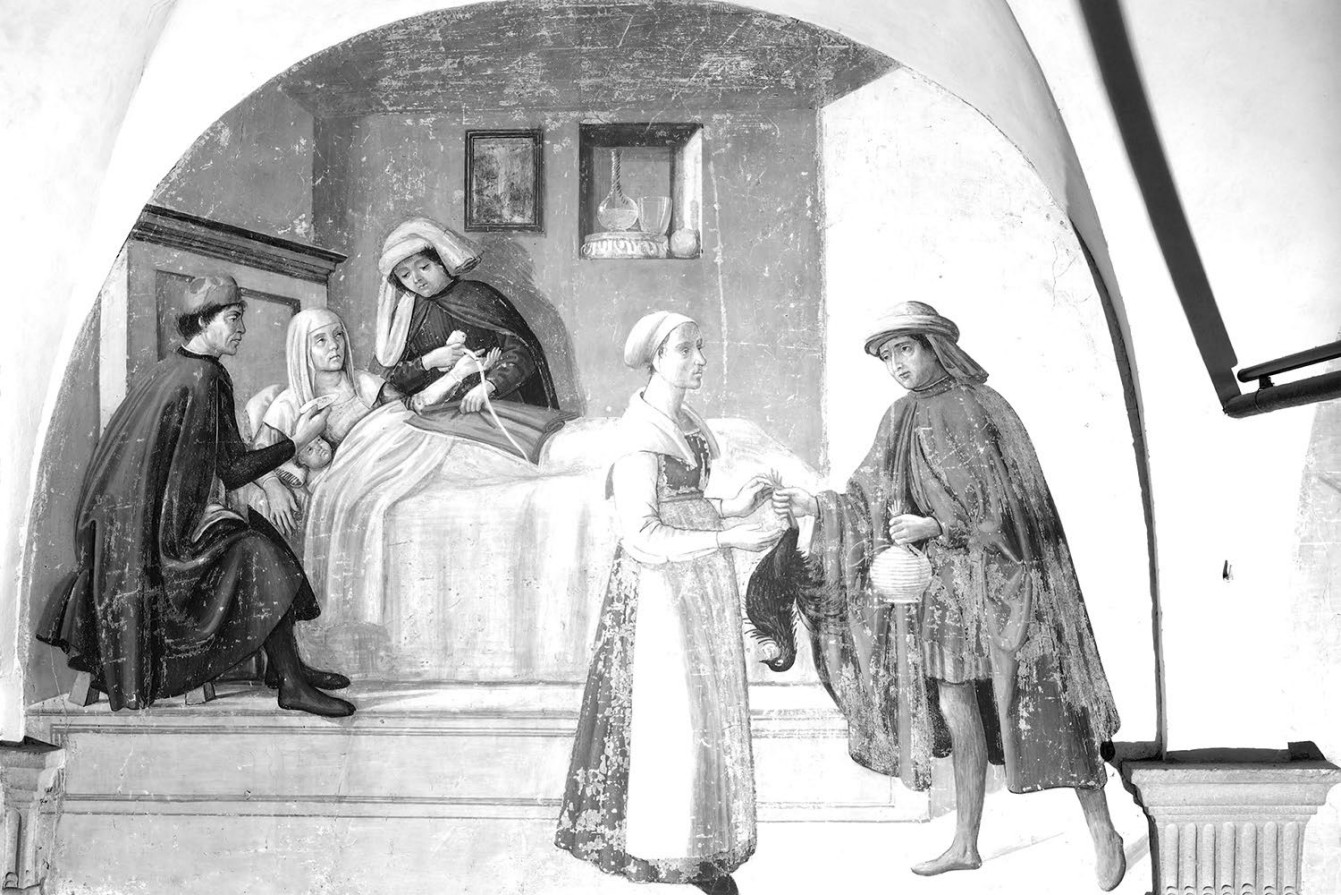
The entire lunette imaged with NIR photography (750–1000 nm).

**Figure 4:**
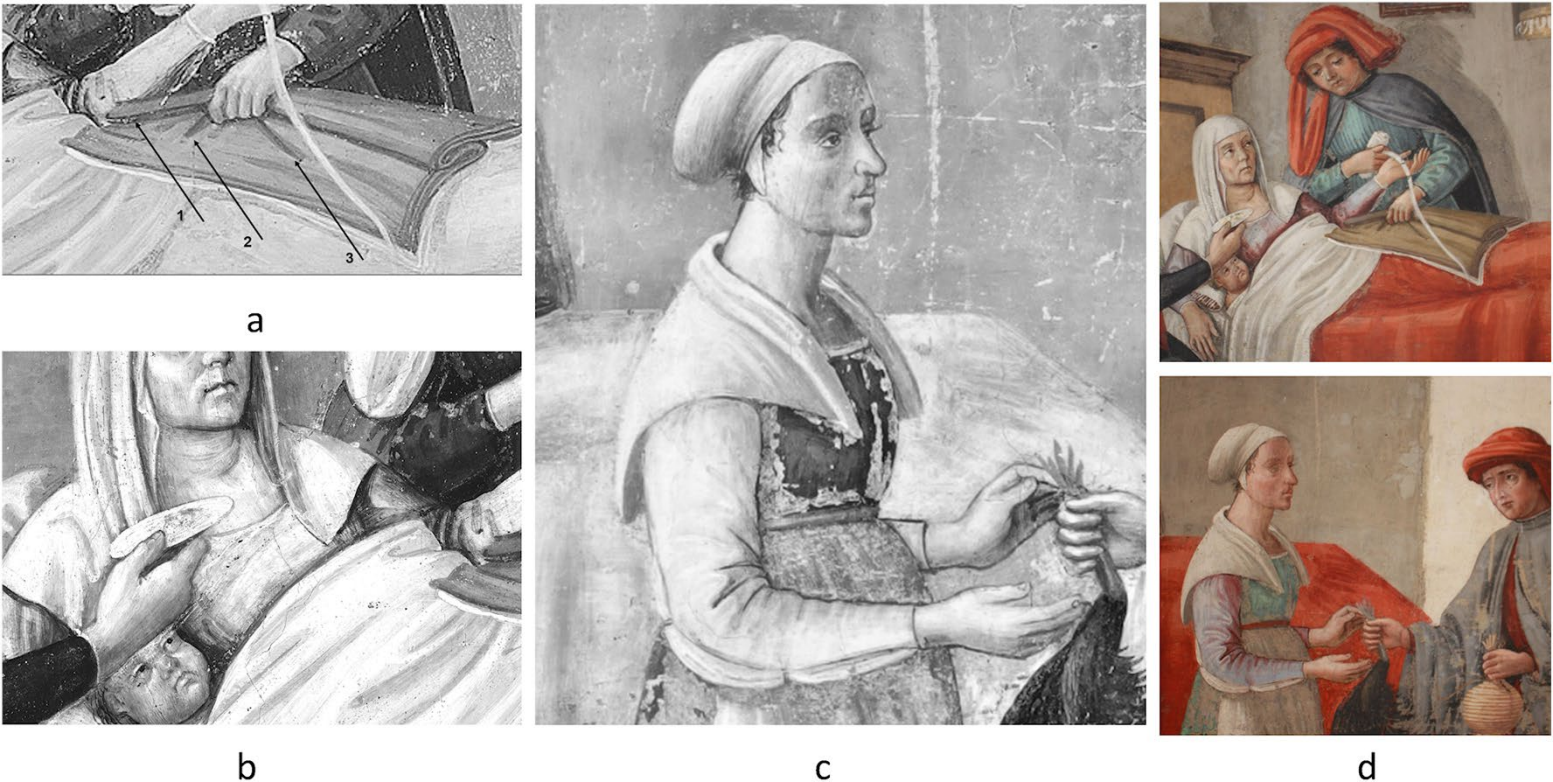
NIR photographs (750–1000 nm) of the details. (**a**) The arrows by the man’s hand indicate the ends of the appendages of the medical instrument. (**b**) The details of the bowl over the mother and child. (**c**) Detail of the standing woman, with visible underdrawings in the contours of visage details and of the sleeves. (**d**) Colour digital photographs. Details of areas of interest.

The full-frame of the painting was preliminarily acquired using the Near Infrared (NIR) photography technique in order to locate the possible presence of underdrawings, *pentimenti* or hidden traits through the entire surface, as well as to visualise the state of the conservation of the pictorial film (Fig. 3). The NIR photograph revealed diffusely spread gaps in the original pictorial film that had been re-integrated by restorations. Mapping these flaked areas could help to locate the original parts of the painting. Overall, the NIR image did not show any evidence in the drawing traits suggesting changes from what is visible today. To get further insight into the underdrawings, NIR images of selected details were acquired at closer distances (0.50 m), in order to increase the quality of details in the final image. As shown in Fig. 4, depending on the investigated areas of the painting, different execution techniques could be detected. No sharp contours were recognisable in some zones where the main characters are depicted, such as the area of the hands of the Buonomo (Fig. 4a), nor the visage of the lying woman, the baby, nor the small cup hand of the second Buonomo handling (Fig. 4b). Marked traits of preparatory drawing were visible in other figures, such as the standing woman (Fig. 4c), by the sharpness of her long eyelashes and the contours of the sleeve. It should be noted that the visibility of these traits in the NIR images proved the efficacy of the technique in capturing the underneath drawings, whenever present, despite the limited spectral range of the NIR photographic camera (850–1000 nm). The parts where no underdrawings could be seen were likely executed with different artistic techniques. Overall, a stylistic variability was observed in the preparatory drawings through the painting surface, with a lesser artistic quality on the minor characters. This is fully consistent with findings on other wall paintings attributed to the same artistic workshop^44^.

The NIR pictures were comparatively evaluated with the corresponding visible images. In the NIR photography of the details of the man hands no underdrawings deviating from the final composition could be evidenced (Fig. 4a). However, the contours of the object in man’s hand appeared sharper than in the visible picture (Fig. 4d), indicating that the current colours might be misleading. In the NIR image the Buonomo seems to be grasping a three-sided shadow, an object consisting of a main body with a square end (no. 1), and two other appendages (no. 2 and no. 3) orientated in two directions. While these shapes appear today in the visible painting as folds of the blanket, their sharper contours in the NIR suggest that originally they could be something different. If it was a bloodletting scene, then this would be a thumb lancet.

**Figure 5:**
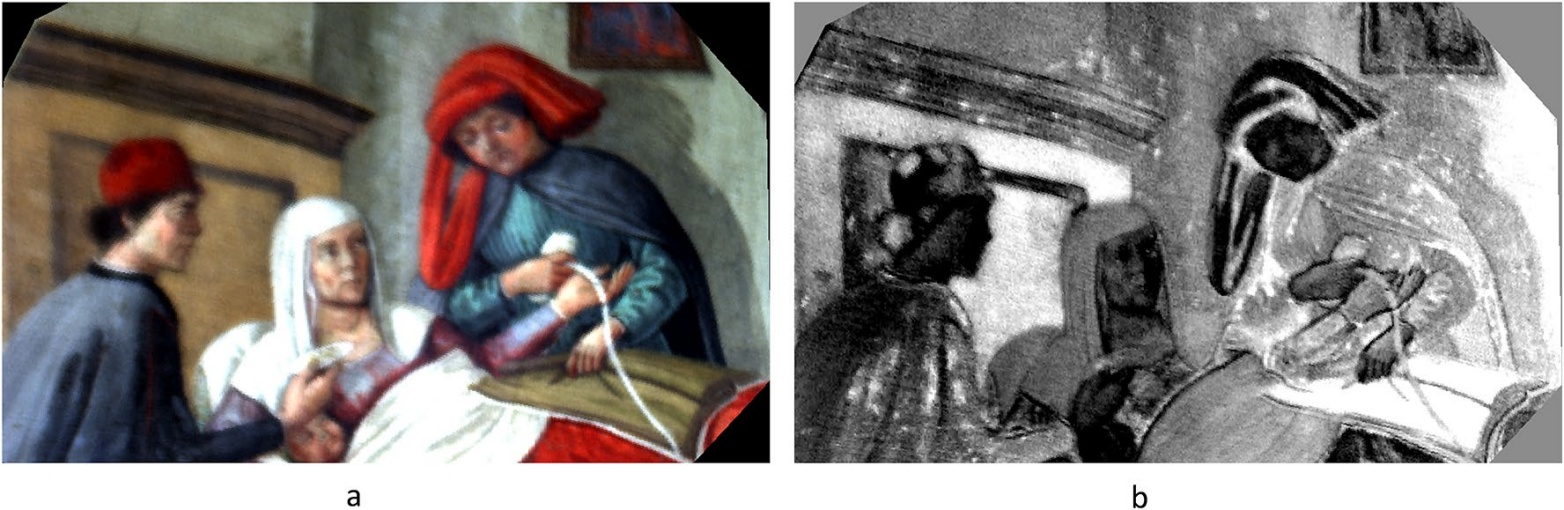
PCA elaboration of the HSI data-cube in the 420–900 nm range. (**a**) Detail of the rectified RGB imaged reconstructed from the HSI data-cube. (**b**) Detail of the rectified PC7 image.

**Figure 6:**
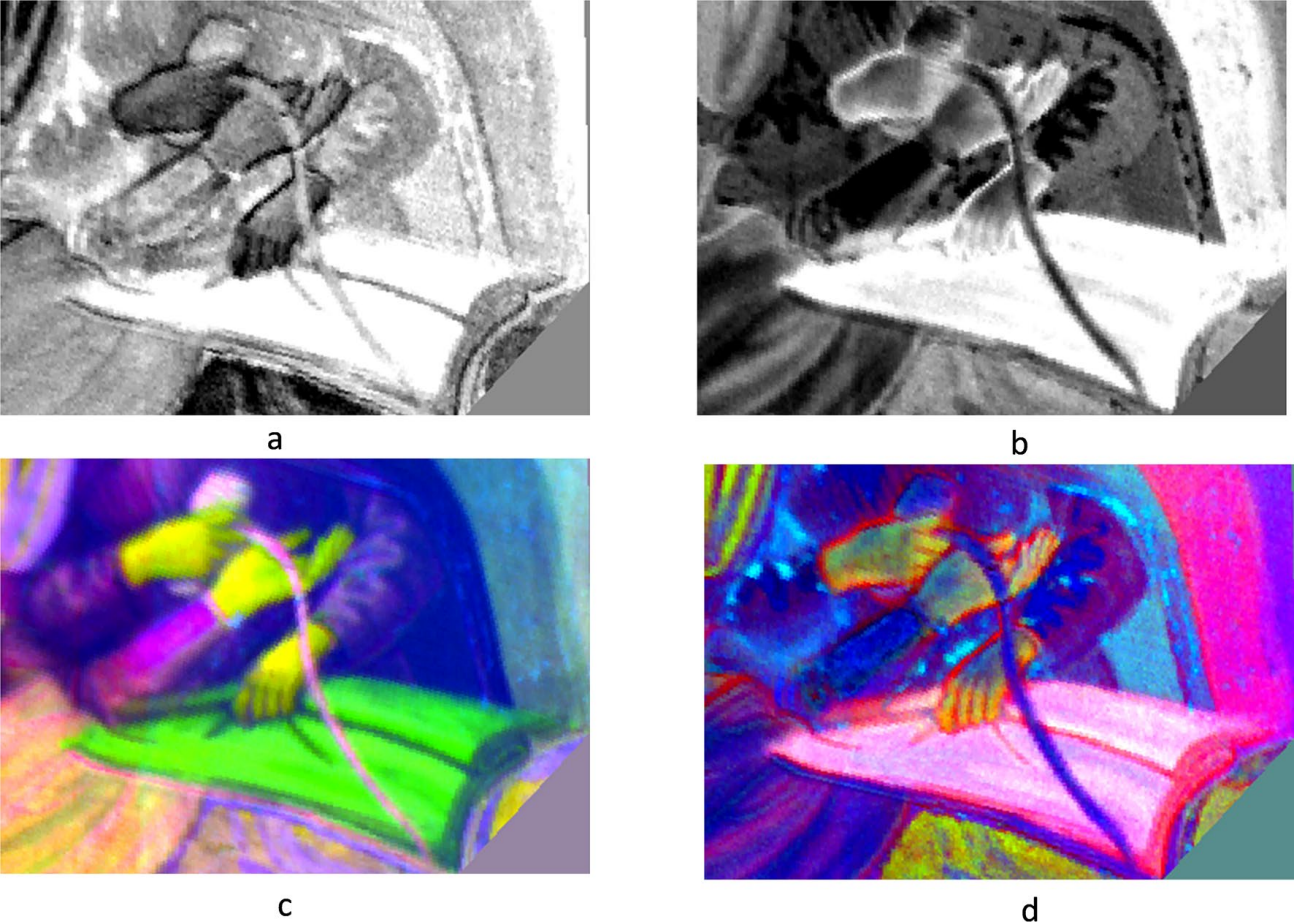
Visualisation of the Buonomo’s hands through HSI elaborated images. (**a**) Detail of the PC7 rectified image. (**b**) Detail of the PC3 rectified image. (**c**) Detail of the false colour image rectified image obtained as MNF2, MNF3 and MNF4 in the RGB channels. (**d**) Detail of the false colour image rectified image obtained as PC3, PC5 and PC7 in the RGB channels.

To strengthen this hypothesis, the HSI images acquired in the Visible and Near Infrared range (VNIR) were analysed. Selected zones of the painting were acquired by changing the HSI camera position, focussing on the regions of interest, and particularly on the men standing by the lying woman. Albeit geometrically distorted and in low resolution, the elaborated HSI data highlighted some differences with respect to the visible painting, that could not otherwise be detected. To reduce the geometrical distortions in HSI data and facilitate the comparison among image-data acquired with different techniques, the HSI images were preliminary registered and rectified (see Section “Data processing”). To enhance the presence of any inhomogeneities in the pictorial film or any features which could not be appreciated by naked eye, Principal Components Analysis (PCA) and Minimum Noise Fraction (MNF) methods were applied to the HSI reflectographic sequences. Figure 5 shows a detail in the rectified RGB image from the HSI data-cube (Fig. 5a) and along with the corresponding area in the PC 7 image (Fig. 5b). As can be noticed in the grey-scale PC7 image, here the blanket appears as flattened, and the object held by the man’s left hand is distinguishable from the blanket folders. The zoom of the area of interest of image PC7 (Fig. 6a) shows that it could be a bulky tool, consisting of three parts, and the length of the grip seems to be shorter than in the final version. The PC3 image, where different features of the scene are emphasised in grey-scale, (Fig. 6b) also shows that originally the grasp seemed to be different. In order to investigate this clue further, Minimum Noise Fraction (MNF), which is an algorithm more effective than PCA in denoising task, was also applied to the HSI data-set^45^. This further analysis confirmed the previous results, as shown in the false colour image shown in Fig. 6c. Even this visualisation, which was obtained by combining the MNF2, MNF3 and MNF4 in the RGB channels, highlighted a three-sided bulky object with the grasp featuring a dark sign suggesting some kind of change. The same effect is also emphasised by the false colour image obtained from PCs combination (PC3, PC5 PC7) (Fig. 6d). Summarising, although the poor spatial definition of the HSI images makes them inconclusive, all the elaborations provided consistent results, which supported the hypothesis.

**Figure 7:**
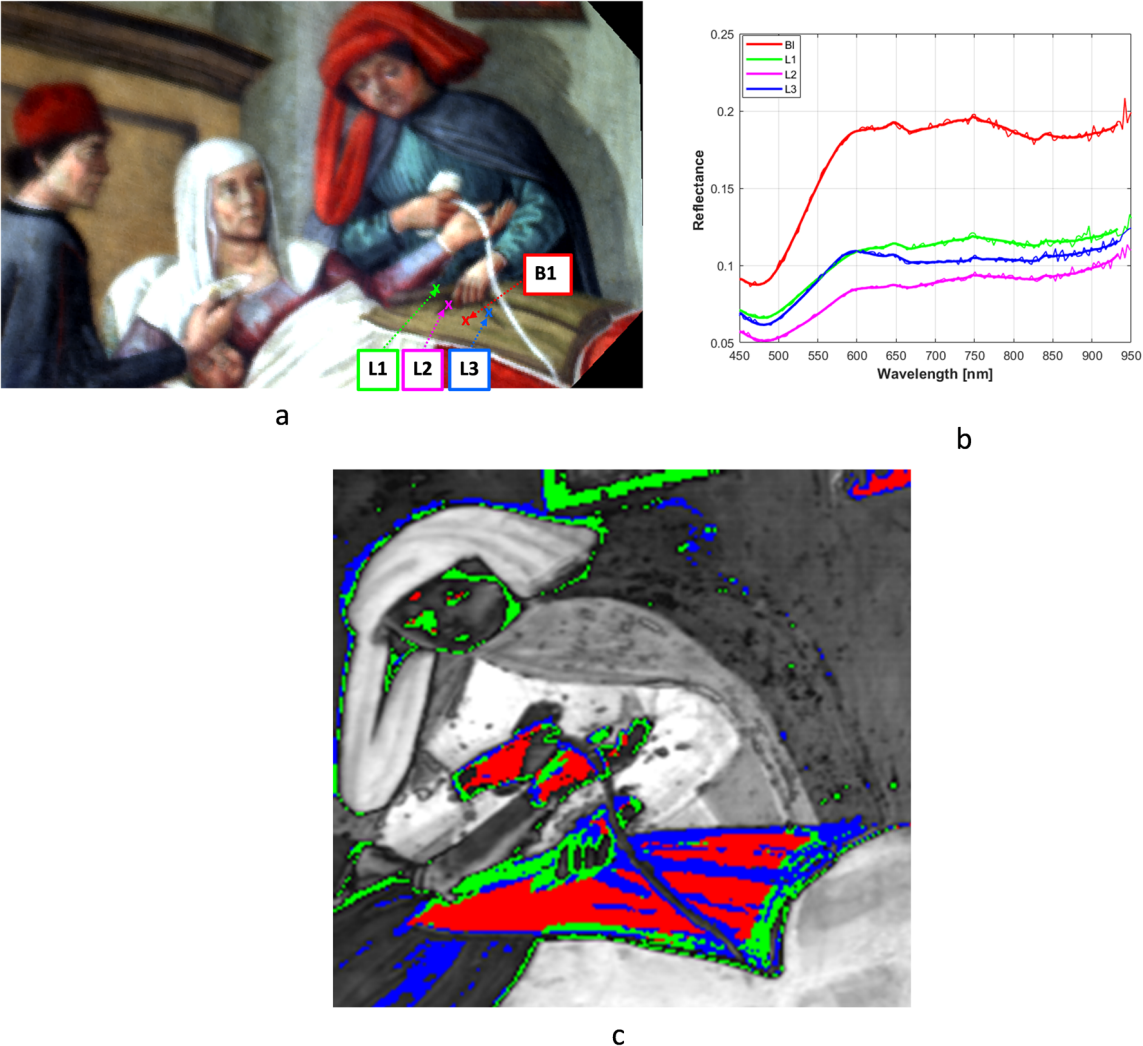
Spectroscopic analysis of the HSI data-cube in the 420–900 nm range. (**a**) Positions of the selected points for extracting spectra from the HSI-data. (**b**) Reflectance spectra extracted at the selected points shown in a). (c) SAM classification map based on the reflectance spectra in b) used as endmembers.

Therefore further investigations were made by analysing the spectroscopic HSI-data. It might be expected that the similar appearance, the pigments mixtures used in the areas which supposedly corresponded to the object in the original version, are somehow different from those used to depict the other parts the blanket. Therefore, the HSI data-cube was exploited to extract reflectance spectra from selected points, namely from the parts of the three-sided shadow in the man’s hand and from the background. The locations of these points are given in Fig. 7a and their VNIR reflectance spectra are reported in Fig. 7b. Despite a poor spectral quality, the markers of Fe(III)-based earth pigments could be clearly recognised in the four spectra, which all feature the characteristics absorption bands of hematite, that is the strong absorption band attributed to the Fe–O bond charge transfer transition and centred around 470–480 nm, and two further absorption bands in the 660–670 nm and 840-850 nm ranges, respectively, which are attributed to the Fe3 + ion ligand field transitions^46^. On the other hand, in the visible picture (Fig. 7a) it is noticeable that the selected points appear as different shades of a same brown colour and, expectedly, their spectral behaviours are similar in the 400–900 nm range, which is dominated by the spectral features of materials at the outer surface. It can be noted that the L1 and L2 spectra do not differ apart the intensity, showing that they were depicted with the same mixtures. A slight variability is anyway observed among the reflectance curves of the background (B1), the L1 and L2 sub-set, and the L3, suggesting a possible change in the respective pigments mixtures and paints thickness. In order to visualise their original distributions over the painting surface better, the Spectral Angle Mapping (SAM) classification algorithm was applied to the HSI-data. The four spectra in Fig. 7b were used as endmembers and the classification map reported in Fig. 7c was obtained. Despite a poor spatial resolution, the map in Fig. 7c highlights the net shape of the object in the man’s hand, as it was mainly depicted with pigment type L2 (green pixels), while the folds of the blanket seem executed with another pigment L3 (blue pixels) and appear as they were stacked from the blanket (red pixels). In the present case, the SAM classificator evidenced the grip and the first appendage of the object, while the third termination remains partly undistinguished in the map.

**Figure 8:**
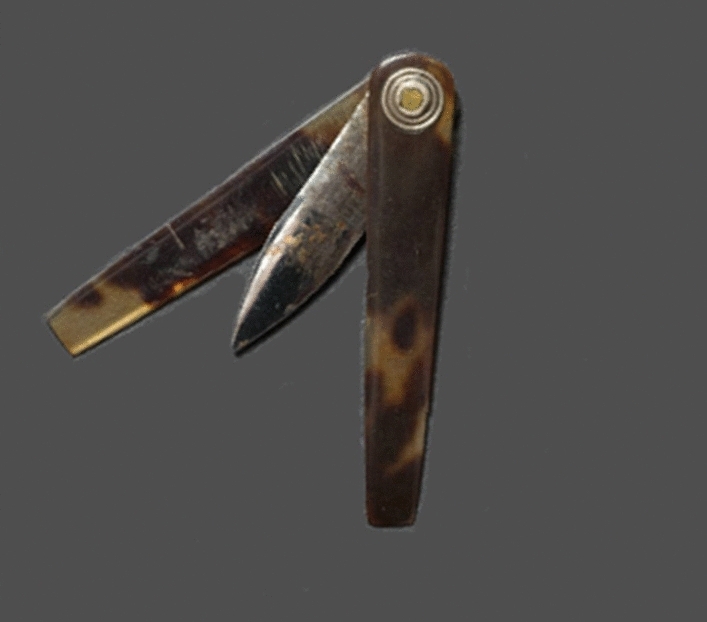
Photograph of a thumb lancet from the eighteenth century showing the folding tortoiseshell handles and the blade [Adapted from photograph of auction item www.worthpoint.com in public domain, no copyright found].

All these elaborations are consistent with the hypothesis that the observed object in the man’s hand was a thumb lancet with its typical shape (Fig. 8) and its purpose was for bloodletting^47^.

**Figure 9:**
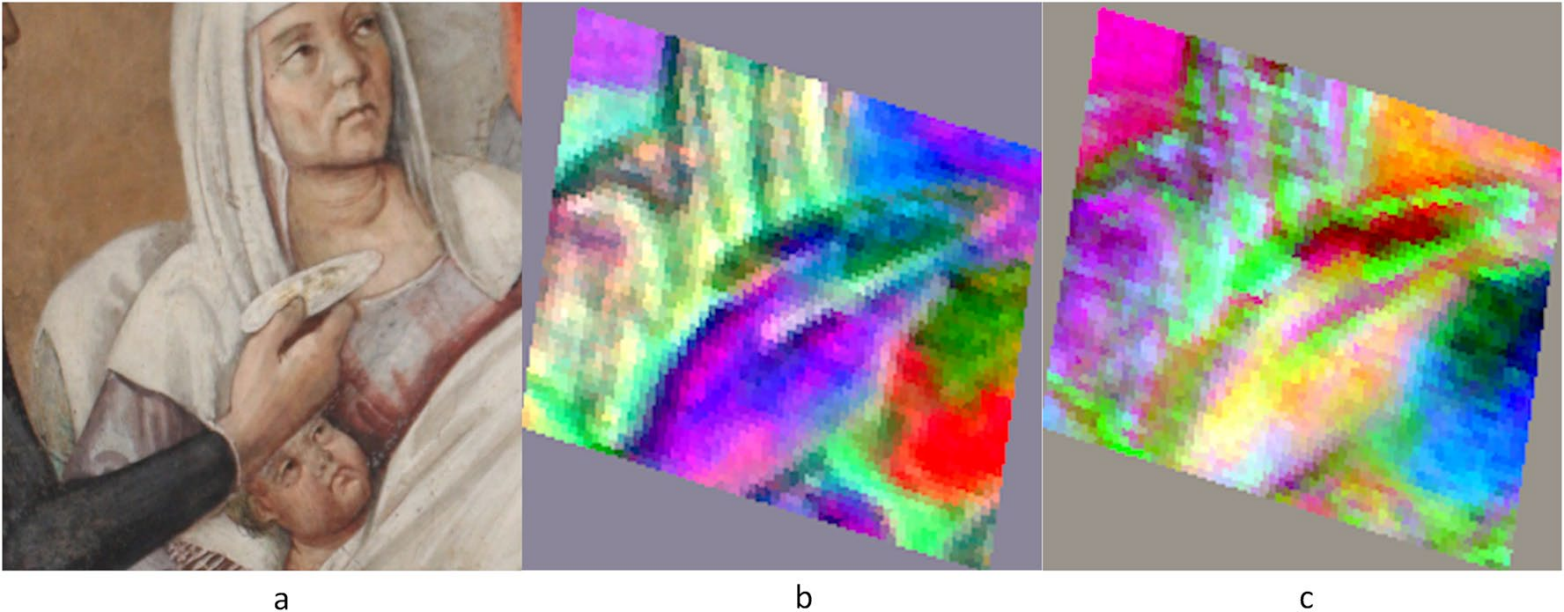
HSI data-analysis in the 420–900 nm range. (**a**) Detail of the RGB high-resolution image. (**b**) Rectified false colour image obtained as PC2, PC3 and PC4 in RGB channels. (**c**) Rectified false colour image obtained as PC3, PC5 and PC4 in RGB channels.

For the small plate in the hand of the second man, PCA analysis has been applied to a region of interest (ROI) in order to enhance its original appearance. Figure 9a is the detail of visible high-resolution photograph is compared with Fig. 9b–c the false colour images obtained by using rectified PC images. In Fig. 9b with PC4, PC3 and PC2 are used in the RGB channels, and in Fig. 9c the used combination is PC3, PC5 and PC4. Both the false colour maps highlight a concavity, today attenuated by the white paint layer that could indicate a bowl rather than a plate.

The hypothesis that the scene can be explained as a bloodletting intervention is technically consistent with other small details in the representation. Namely, the unrolled bandage is a tourniquet; the rolled part used subsequently to apply pressure to stop the vein bleeding. The bowl collects the blood. The woman is holding out her left hand with the bandage lying across the palm suggesting that this is the tourniquet site.

A further historical detail to consider is that the Buonomini worked in pairs, one for each of the six districts of Florence. They kept detailed accounts of their work which included payments to physicians and medicines, and general medical assistance if beneficiaries were sick, disabled, or in childbirth^48^. Bloodletting was a skill learned by apprenticeship but no formal recognition was required nor association with a particular Guild^48^ Given that the picture shows a Buonomo bloodletting, this suggests that some of them developed the necessary competencies. The practice would always be under the guidance of a physician so it is possible that the sitting man in the picture is one, but in expensive civilian clothes. The other standing man who is giving food and drink, who wears similar clothing to the phlebotomist, is then the second Buonomo.

## Conclusions

The combination of two stand-alone imaging techniques such as Vis–NIR Hyperspectral Imaging with Near Infrared photography made it possible to make visible some lost details in the painting “Visit to the Sick” in the Buonomini Oratory in Florence. The enhanced visualisation of objects held in the hands suggest a new interpretation of the painting whereby the scene does not represent a simple visit to give gifts and comfort to a *post partum* woman, but a medical treatment.

The hypothesis is therefore that the mural is a bloodletting scene for a mother suffering from either *postpartum* depression or puerperal fever. The standing Buonomo is a trained phlebotomist. He is supervised by a physician who decides when and how much blood is to be drained. The cloth is the bandage/tourniquet used to define the *salvatella*, and the site for treating melancholia and fevers. The other standing man is the second Buonomo who is bringing alms of food and drink. At the time the mural was painted the thumb lancet was a new medical instrument, whose diffusion is scarcely documented. Archival research indicates that it was introduced in 15th Century in Italy^49^. The painting of *Visit to the Sick* may be the earliest pictorial representation of this device.

## Methods

Details of the methodology of the archival research have been reported^50^.

The difficult accessibility of the mural raised several technical challenges in capturing images capable of some iconographic elements. Portable HSI in the 400–1000 nm range and Near-InfraRed (NIR) Photographic Reflectography were combined to inspect the painting in remote sensing mode, to cover the Visible and the Near Infrared spectral ranges at adequate spectral and spatial resolutions. Measurements were performed in August 2022, during the opening hours. The room being a site of historical interest cumbersome scaffolding was considered incompatible. A system of tripods was used to mount the equipment (cameras and lights). The cameras were suitably positioned in order to acquire different aspects at variable distances and orientations from the painting surface. The same illumination sources kept in a fixed position served both imaging techniques, thus reducing the in-situ gear needed.

Using tripods meant sub-optimal positioning of cameras and lights with respect to the target surface. This resulted in both non-uniform illumination of the target areas and geometrical deformation of the acquired image. These problems can affect the quality of final data; nevertheless, in the case under examination the output images do not impair the possibility of singling out details of the painting that are not appreciable by visual inspection. The comparability between output images acquired under different experimental conditions and using different devices was ensured using rectification and registration algorithms in post-processing phase, as detailed in Sect. “Data processing”.

### Lighting system

A unique lighting system was used for both HSI and Near Infrared Photography acquisitions. A single reflector mod. ARRILTE 750 Plus with a tungsten halogen lamp colour temperature 3200 K. The light source was mounted on a tripod, it was orientated at 45° to the normal at the surface and placed at a distance of about 8 m from the centre of the painting (Fig. 1). This configuration guaranteed uniform washing lighting on the entire painting target surface. Aside from addressing compatibility with the room’s logistic obstacles, e.g. avoiding shadows from architectonic elements, setting-up of lighting system had to be a compromise between other contrasting requirements. The intensity of the incident radiation at the target surface had to guarantee proper signal to noise ratio, in particular in spectroscopic measurements, such as HSI. At the same time, any risks of photo-induced damages on the painting surface had to be prevented, thus minimising the exposure of the target to the light. This issue was managed by preliminarily checking the illuminance levels at the target surface and then, during the entire measurements campaign, by keeping the cumulative light-exposures (expressed in lux x hours) within the recommended levels according to the European Standard for lighting museum objects^51^.

### Near-InfraRed Photography

Infrared Reflectography (IRR) includes a class of techniques generally used to detect underdrawings^52,53^ with several options involving variable actions that depend on the sensors, optics and experimental setups^54–57^. As a simplified version of IRR, Near-InfraRed (NIR) Photography uses normal digital cameras modified to exploit the 750–1100 nm region that is the tail of the sensitivity range of commercial CCD Silicon detectors. Despite limited coverage, NIR Photography is effective for the preliminary inspection of the layers beneath the painted surfaces^58,59^. Therefore, it was selected here because it is lightweight and highly versatile. The measurements were performed using a modified digital camera mod. Canon EOS RP, full-spectrum converted^60^ and equipped with Canon 50 mm f/1.8 STM objective lens, and a full frame 26.2 Megapixel CMOS sensor, 35.9 × 24 mm size. The modification consisted in the removal of the production camera’s low-pass filter and replacement with an IR pass filter at 850 nm. The matrix sensor guaranteed excellent image quality, even under remote shooting conditions. NIR images were acquired with following parameters settings: Shutter speed: 1/8; Aperture: 8.0; Field of view: 39.5°; Image pixel resolution: 6240 × 4160. The camera was mounted on a tripod, which was positioned at different distances to shoot pictures of variable size and different spatial resolutions. The full scene (Fig. 3) was acquired from a distance of 3.5 m from the centre of the fresco, resulting in a spatial definition of approximately 25pixel/cm. The images of details (Fig. 4a–c) were acquired at closer distance, placing the camera at 50 cm from the target areas. These images feature spatial definitions between 90 pixel/cm and 170pixel/cm.

### Portable VNIR hyperspectral imaging

HSI provides a sequence of spectrally contiguous reflectographic images acquired throughout the sensitivity interval of the HSI camera. The output is a data-set (image cube), in which each pixel (point) of the framed image is associated with a reflectance spectrum^61^. The HSI data are big size sets embedding rich but redundant information. The HSI data in the Visible range are used for reconstruction of colour RGB images, while the spectral images in the NIR range enable detection of features below the painted surfaces, such as underdrawings.

HSI data analysis is performed with suites of algorithms for data-reduction and multivariate analysis, selected on the basis of the specific applications. As outputs, set of new images are generated, which perfectly overlap with the RGB colour images of the framed scene, highlighting elements which are not evident on visual inspection, such as paint layer inhomogeneities, retouches, re-paintings, or other anomalies on the surface. For these reasons the HSI technique is therefore ideal for the investigation of paintings and polychrome artworks, since it is non-invasive and provides compositional information on pigments and artists’ materials on the entire surface, while allowing production of multiple elaborated images (maps, false colour and grey-scale images, etc.) which document the state of conservation of the painting and its concealed features.

In the San Martino Oratory, a portable HSI camera push broom type mod. Specim IQ was used. The camera is compact (207 × 91 × 74 mm) and light (1.3 kg). The nominal spectral range is 400–1000 nm, with 7 nm spectral resolution. The HSI data sets include 204 bands (spectral images). The sensor is a CMOS type, 512 × 512 pixels. The optical objective works at variable distances from the target, from a few centimetres to infinitity. The F/number of the optical system at the sensor is 1.7. The Field of View is 31°. In the measurement campaign the optimal distance was established on the basis of operational needs, considering that as the distance increases, the framed area increases but reduces the spatial resolution, and vice versa. In order to maximise the visibility of the details in the areas of interest, different image-cubes were acquired varying the position and orientation of the HSI camera. A high-elevation tripod was used to mount the camera, enabling positioning in front of the target areas within the lunette. Eleven data-sets were acquired, which framed different sizes and portion of the painting. In order to get HSI images which could be visually compared with the NIR photographs, the camera position was varied in a range of distances between 3.5 and 1.5 m from the wall painting. In comparison with the NIR photographs the acquired HSI images were of poorer quality, featuring spatial definitions which ranged between 4 and 2 cm/pixel, depending on the set-up. The configuration resulted in poor detail quality of HSI images, but nevertheless data could be effectively exploited to extract spectroscopic information, which provided evidence of materials differences with respect to the visible image.

White reference calibration was performed using the “simultaneous” acquisition-mode, which consisted of including the white target, a certified 99% Reflectance Spectralon plate, within the acquisition frame. This choice minimises the undesired effects due to the unevenness of illumination and optimises the signal-to-noise ratio in spectral data. The drawback is that positioning the white target in the desired places can be difficult practically, since any contact with the painting should be avoided. In the examined case, the Spectralon plate was mounted on a high elevation tripod, so to enable its acquisition within the selected area to image, without coming in touch with the painting surface.

### Data processing

Data processing of HSI data-cubes acquired with Specim IQ was performed using the ENVI© software^62^.

The HSI camera set-up was set to optimise the acquisition of the ROIs (the objects in the Buonomini hands) at acceptable spatial and spectral resolutions, but at the cost of geometrical distortions. The HSI were therefore rectified in the post-processing phase. With this aim, the HSI data-cubes and their elaborated outputs (PCA; MNF, etc.) were registered by using the corresponding spatial ROIs in the NIR and Visible RGB high resolution photographs acquired with the Canon camera. The function image-to-image of ENVI Classic’s menu was used, with selection Ground Control Points (GCPs). This workflow enables geometrical alignment of two images featuring different viewing geometry. In the examined case, the NIR or RGB photographs (featuring higher resolutions and no distortions) were set as base images for registration and warping of HSI-data. Due to the different extension and spatial resolution of the data to be fused, it was decided to optimise correction of the ROI rather than the entire scene. The best results were obtained using the Delaunay triangulation method. The algorithms here applied were: principal component analysis (PCA), minimum noise fraction (MNF) and the classification method Spectral Angle Mapping (SAM).

Any possible material inhomogeneities or hidden feature were investigated using PCA and MNF methods. PCA and MNF are statistical techniques of dimensionality reduction. They were applied to the Vis–NIR HSI data sets to facilitate the visualisation of possible hidden features of the painting that could be not visible to the naked eye. PCA and MNF algorithms stress the variability of the spectral data-set and eliminate the redundant contents. When used to process HSI data acquired on polychrome paintings, these methods emphasise the presence of dishomogeneities of the paint films (such as non-original retouches, variations of thickness, degraded areas etc.) in spite of their similar appearance. In the present study the PCA and MNF algorithms were applied to the pre-treated HSI data-cubes. Pretreatment included spectral cut at the edges of range to exclude the noisy contributions. The resulting data-set included 170 spectral bands in the 420-920 nm range. PCA transformation was made using Covariance Matrix; in the reported results the first 10 PC images were retained, which summarised the 99.98% of the data variance. Also the MNF algorithm was applied to the same pre-treated data-set. A sequence of 10 MNF images was retained. MNF bands were suitably combined in RGB channels to obtain false colour images.

Pre-processing before applying SAM algorithm included smoothing of the spectra by means of inverse PCA transformation to the HSI data, and subsequent sub-setting of data in the 420-930 nm range. The SAM algorithm was applied to the entire spatial area (no spatial sub-sampling) of the HSI data-cube. Endmembers spectra were extracted by the pre-treated HSI data-cube according to the criteria reported in sect. “Results and discussion” the Maximum Angle (MA) threshold was set to 0.5 radians to obtain the map in Fig. 7c.

The NIR and RGB image-files which were acquired with the Canon EOS digital camera were treated with XnView MP free software^63^.

## Data Availability

Raw data can be obtained on request from Professor Cucci; email address: c.cucci@ifac.cnr.it.
